# Supplementary material for: Chromothripsis during telomere crisis is independent of NHEJ, and consistent with a replicative origin
Source: Genome Res. 2019 May;29(5):737–49. doi: 10.1101/gr.240705.118 (PMC6499312; doi:10.1101/gr.240705.118)
Supplement: Supplemental Material [file supp_gr.240705.118_Supplemental_file_1.zip › contigs/annotated_contigs/DB79/contig.2.DB79_length_376_mean_cov_5.96808510638.docx]

**DB79_length_376_mean_cov_5.96808510638**

```
CATAGTTTCCGAATGGAATGCATGTATTTGTAGGCCAATGCAGAGAAAGAATTTTTGACAGTCTTCTGAGCAGTCTCCTTGAAACTAAA
 >chr22:18209042-18209320 - E=3e-145 p=3e-02
AAATCCTAAGTTAATATACATTCAGTGTAATTCACACACAGGCTGAGAGCATCCTAATCTGAAAACCTGAAATCTGAAATGATCCAAAA

TCTGAGGTTTTTTTTTTTTTAAGATGGAGTCTTGCTCTGTCTCCCAGGCTAGAGTGCAGTGGCACAATCTCAGCTCACTGCAACCTCCG

CGTCC|GGAGGAGCGGCGAGTCGGGTACGTGAGCCGGCCAGGGAGCAGGGCTCGGAGGCCGGCACA|ACCACACCAGGCTAATTTTTGT
                                                                  >chr4:17554562-175546
ATTTTTAGGAGAGATGAGATTT06 - E=5e-12
```
